# Association between peripheral blood serum phenylalanine to tyrosine ratio and the risk of moyamoya disease: a case-control study

**DOI:** 10.3389/fneur.2025.1554697

**Published:** 2025-04-03

**Authors:** Shixiong Lei, Mengnan Wang, Jiali Pan, Daming Wang, Peicong Ge, Dong Zhang

**Affiliations:** ^1^Department of Neurosurgery, Beijing Hospital, National Center of Gerontology, Institute of Geriatric Medicine, Chinese Academy of Medical Sciences, Beijing, China; ^2^Graduate School of Peking Union Medical College, Beijing, China; ^3^Department of Neurology, Sanbo Brain Hospital, Capital Medical University, Beijing, China; ^4^Department of Neurosurgery, Beijing Tiantan Hospital, Capital Medical University, Beijing, China; ^5^China National Clinical Research Center for Neurological Diseases, Beijing, China

**Keywords:** phenylalanine to tyrosine ratio, moyamoya disease, biomarker, circle of Willis, risk factor

## Abstract

**Background and aims:**

Phenylalanine (Phe) and its metabolite tyrosine (Tyr) have been shown to play an important role in the mechanisms and development of cardiovascular and cerebrovascular disease, and its ratio (Phe/Tyr) has been suggested to be an important indicator of inflammation. It was uncertain whether Phe/Tyr is associated with higher risk of MMD. Therefore, we conducted this study to evaluate the relationship between Phe/Tyr and the risk of MMD and its subtypes.

**Methods:**

A total of 360 adult MMD patients and 89 age-matched healthy controls (HCs) were consecutively recruited for this prospective study. We measured peripheral blood serum Phe and Tyr levels in all participants to analyze the association between Phe/Tyr and the risk of MMD and its subtypes.

**Results:**

Serum Phe/Tyr was significantly higher in MMD patients and their subtypes than in HCs (*p* < 0.01). After adjusting for traditional risk factors, Phe/Tyr was positively associated with the risk of MMD (OR: 14.035, 95%CI: 2.784–70.748, *p* = 0.001). When Phe/Tyr was assessed in quartile subgroups, the third quartile (Q3) and fourth quartile (Q4) subgroups of Phe/Tyr had a significantly increased risk of MMD compared to the first quartile (Q3, OR: 2.019, 95%CI: 1.066–3.824, *p* = 0.031; Q4, OR: 2.887, 95%CI: 1.446–5.765, *p* = 0.003). The risk of MMD subtypes also increased with elevated Phe/Tyr level. Meanwhile, the addition of Phe/Tyr to conventional risk factors could significantly improve the risk prediction for MMD.

**Conclusion:**

In this study, the risk of MMD increased with elevated Phe/Tyr, suggesting that peripheral blood serum Phe/Tyr may be a valuable predictive biomarker of adult MMD.

## 1 Introduction

Moyamoya disease (MMD) is a cerebrovascular disease characterized by progressive narrowing of the end portion of the internal carotid arteries and the formation of a fragile smoke-like network at the base of the brain (1). There are clear geographical differences in the incidence of MMD, which is more common in Japan, China, Korea and southeastern Asian countries (2). The prevailing trigger in the pathogenesis of MMD was thought to be genetic susceptibility (3). The discovery of RNF213 as a major susceptibility gene for MMD in East Asian populations opened new avenues for studying disease mechanisms and potential therapeutic targets (4–6). In addition, several traditional modifiable risk factors have been shown to be associated with MMD in our previous study (7) The exact pathogenesis of MMD is still unclear but it is more likely to be a multifactorial disease related to genetic, inflammation, immunne, and other factors (4, 8). However, not all cases can be explained by traditional risk factors, so it is essential to investigate the pathogenesis of MMD and identify new risk factors involved in its progression.

Phenylalanine (Phe) plays important roles in many physiological and pathological processes of the nervous system and other systemic diseases (9, 10). Tyrosine is an important metabolite of phenylalanine and a precursor for the synthesis of catecholamines and neurotransmitters. Fluctuating levels of Phe and Tyr in many disease states lead to changes in the Phe/Tyr ratio. Previous studies have shown that impaired conversion of phenylalanine to tyrosine is associated with chronic renal failure (11). Phe/Tyr ratio is also effective and applicable in predicting premotor Parkinson’s disease (12). Phe/Tyr is significantly elevated in the acute phase of acute ischemic stroke and is considered a potential biomarker (13). The aim of this study was to clarify the association between Phe/Tyr ratio and the risk of MMD and to help identify new potential biomarkers.

## 2 Materials and methods

### 2.1 Study participants

**Figure 1:**
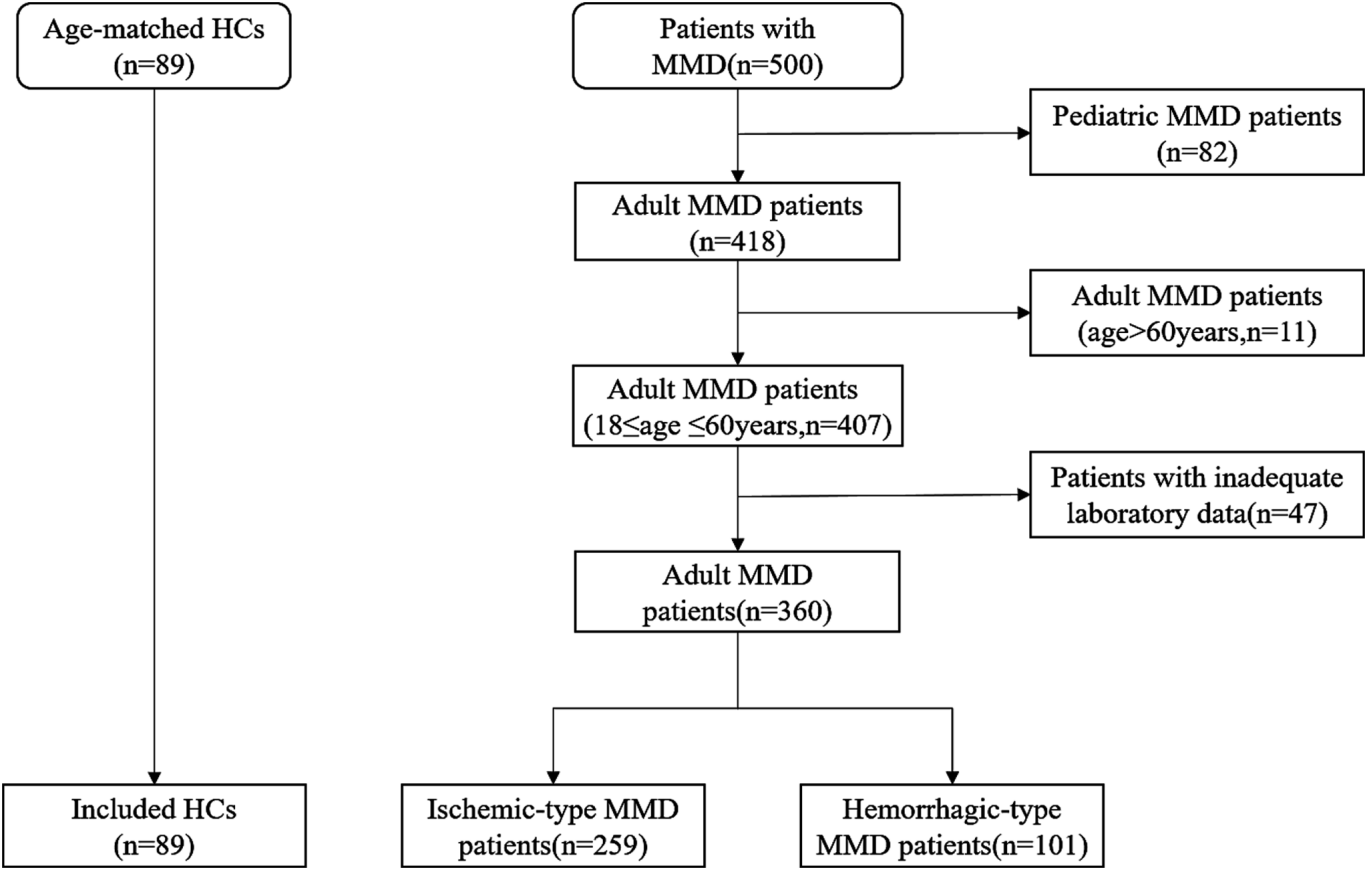
Flow chart of the study participants. HCs, healthy controls; MMD, moyamoya disease.

This prospective study continuously recruited 500 patients with MMD who were treated at Beijing Tiantan Hospital from September 2020 to December 2021. All patients must complete digital subtraction angiography (DSA) and be in compliance with the diagnostic criteria of the Japanese guidelines released in 2012 for the diagnosis of MMD. Pediatric patients and elderly patients over 60 years old were excluded. 47 patients were excluded due to lack of Phe and Tyr laboratory data. The flow chart for the inclusion of all study participants is shown in Figure 1. Finally, 360 cases of adult MMD patients were included, including 259 cases of ischemic MMD and 101 cases of hemorrhagic MMD. Eighty-nine age-matched healthy (HCs) people were included in the control group. These healthy participants and their family members have no history of MMD, heart disease or other cerebrovascular diseases. Informed consent to participate in this study was obtained from all participants.

### 2.2 Data collection

We collected the clinical characteristics of all participants on admission, including age, gender, body mass index (BMI), heart rate, and blood pressure, as well as past medical history (hypertension, diabetes, hyperlipidemia, smoking, and alcohol consumption). After a 15-min rest, the participants’ right arm systolic blood pressure (SBP) and diastolic blood pressure (DBP) were measured using a traditional mercury manometer, and heart rate data were recorded using an electrocardiograph. Fasting peripheral blood samples were collected from all participants for routine and biochemical blood tests, and blood samples were collected for Phe and Tyr determination. Serum samples are separated by centrifugation within 1 h and stored in a central laboratory fridge at −80°C until further analysis. Liquid chromatography-mass spectrometry (LC–MS) analysis was used for the quantification of Phe and Tyr. The laboratory technicians were unaware of the baseline characteristics of all patients. Genomic DNA was extracted from 350 patients using the QIA amp blood kit (QIAGEN, Hilden, Germany). We used primers named RNF213-4810F (rs112735431) 5’-GCCCTCCATTTCTAGCACAC-3’ and RNF213-4810R 5’-AGCTGTGGCGAAAGCTTCTA-3’ to detect the RNF213 p.R4810K variant. In total, 64 patients with the RNF213 rare variant p.R4810 k (rs112735431) were found in the 350 sequenced patients. We used the modified Rankin Scale (mRS) to evaluate neurological function status on admission. Suzuki staging was categorized based on the higher grade observed bilaterally. Baseline of all study participants are shown in Table 1.

**Table 1:** Clinical and laboratory characteristics in MMD patients and HCs.

Variables	HC (*n* = 89)	MMD (*n* = 360)	*p-*value	Ischemic (*n* = 259)	*p-*value	Hemorrhagic (*n* = 101)	*p-*value
Age, years, mean ± SD	39.81 ± 11.57	41.60 ± 10.25	0.151	41.53 ± 10.12	0.185	41.78 ± 10.61	0.198
Gender, male, *n* (%)	37 (41.6)	150 (41.7)	0.987	116 (44.8)	0.598	34 (33.7)	0.261
Medical history							
Hypertension, *n* (%)	0 (0.0)	131 (36.4)	<0.001	102 (39.4)	<0.001	29 (28.7)	<0.001
Diabetes, *n* (%)	0 (0.0)	59 (16.4)	<0.001	55 (21.2)	<0.001	4 (4.0)	0.058
Hyperlipidemia, *n* (%)	0 (0.0)	54 (15.0)	<0.001	45 (17.4)	<0.001	9 (8.9)	0.004
Smoking, *n* (%)	2 (2.2)	71 (19.7)	<0.001	54 (20.8)	<0.001	17 (16.8)	0.001
Drinking, *n* (%)	0 (0.0)	42 (11.7)	0.001	34 (13.1)	<0.001	8 (7.9)	0.007
Clinical features, mean ± SD							
Heart rate, bpm	77.79 ± 9.73	78.54 ± 6.42	0.375	78.25 ± 6.59	0.602	79.30 ± 5.92	0.149
SBP, mmHg	123.64 ± 11.77	132.33 ± 12.81	<0.001	133.56 ± 12.84	<0.001	129.16 ± 12.25	0.003
DBP, mmHg	78.46 ± 8.35	81.85 ± 9.33	0.002	82.45 ± 9.42	<0.001	80.32 ± 8.96	0.162
BMI, kg/m^2^	23.94 ± 3.40	25.43 ± 4.51	0.004	25.87 ± 4.57	<0.001	24.28 ± 4.15	0.585
Laboratory examinations, mean ± SD							
WBC count, 10^9^/L	6.09 ± 1.49	7.17 ± 2.08	<0.001	7.26 ± 2.00	<0.001	6.93 ± 2.25	0.004
LY count, 10^9^/L	1.89 ± 0.50	2.01 ± 0.67	0.140	2.07 ± 0.59	0.022	1.83 ± 0.80	0.516
NEUT count, 10^9^/L	3.69 ± 1.32	4.62 ± 1.94	<0.001	4.64 ± 1.80	<0.001	4.59 ± 2.27	0.001
MONO count, 10^9^/L	0.35 ± 0.11	0.38 ± 0.14	0.084	0.39 ± 0.14	0.035	0.36 ± 0.13	0.649
PLT count, 10^9^/L	248.26 ± 59.57	255.72 ± 70.75	0.359	259.01 ± 71.65	0.203	247.30 ± 68.01	0.923
GLU, mmol/L	5.10 ± 0.50	5.53 ± 1.55	0.010	5.71 ± 1.71	<0.001	5.07 ± 0.89	0.888
ALT, g/L	22.00 ± 14.34	27.60 ± 24.32	0.038	28.79 ± 25.43	0.015	24.56 ± 21.04	0.437
AST, g/L	18.99 ± 5.11	19.61 ± 9.26	0.541	19.48 ± 9.19	0.643	19.95 ± 9.48	0.441
ALB, g/L	45.12 ± 2.20	45.25 ± 2.96	0.697	45.28 ± 3.01	0.635	45.16 ± 2.83	0.921
Urea, mmol/L	6.46 ± 10.57	4.70 ± 1.37	0.002	4.67 ± 1.34	0.003	4.79 ± 1.45	0.018
Cr, μmol/L	58.26 ± 15.08	57.05 ± 14.05	0.471	57.30 ± 13.68	0.581	56.41 ± 15.01	0.371
UA, μmol/L	310.61 ± 74.27	315.62 ± 90.23	0.628	322.55 ± 91.02	0.264	297.85 ± 86.06	0.313
TG, mmol/L	1.10 ± 0.73	1.42 ± 1.06	0.006	1.46 ± 1.15	0.003	1.33 ± 0.80	0.116
TC, mmol/L	4.65 ± 0.73	4.23 ± 0.98	<0.001	4.09 ± 0.96	<0.001	4.57 ± 0.94	0.557
HDL- C, mmol/L	1.52 ± 0.29	1.31 ± 0.28	<0.001	1.28 ± 0.28	<0.001	1.38 ± 0.26	<0.001
LDL- C, mmol/L	2.72 ± 0.66	2.44 ± 0.85	0.003	2.31 ± 0.81	<0.001	2.76 ± 0.84	0.750
ApoA, g/L	1.40 ± 0.19	1.30 ± 0.25	<0.001	1.29 ± 0.26	<0.001	1.31 ± 0.24	0.014
ApoB, g/L	0.82 ± 0.20	0.84 ± 0.22	0.488	0.82 ± 0.21	0.954	0.89 ± 0.24	0.032
Homocysteine, μmol/L	11.22 ± 6.27	13.40 ± 6.92	0.007	13.63 ± 7.41	0.004	12.79 ± 5.46	0.112
Phenylalanine, μmol/L	90.79 ± 15.43	95.61 ± 17.88	0.020	95.30 ± 17.10	0.036	96.43 ± 19.82	0.027
Tyrosine, μmol/L	74.76 ± 12.86	73.52 ± 15.11	0.476	73.75 ± 14.78	0.576	72.93 ± 16.01	0.393
Phe/Tyr ratio	1.22 ± 0.18	1.33 ± 0.23	<0.001	1.32 ± 0.23	<0.001	1.34 ± 0.23	0.001
Phe + Tyr, mmol/L	165.55 ± 25.61	0.364	0.364	0.364	0.364	0.364	0.364

### 2.3 Statistical analysis

Continuous variables were expressed as mean standard deviation (SD) and categorical variables as frequencies. Continuous variables were compared between the two groups using T-test or Mann–Whitney U test, and categorical variables were compared using Pearson chi-squared test and Fisher exact test. Kruskal-Wallis test or one-way ANOVA was used to compare groups. Three logistic regression models were used to examine the association between Phe/Tyr and the risk of MMD and its subtypes. The crude model is an unadjusted Phe/Tyr regression model. Model 1 was adjusted for sex, age, HR, SBP, DBP, BMI. Model 2 was further adjusted for white blood cell count (WBC), lymphocyte count (LY), neutrophil count (NEUT), monocyte count (MONO), platelet count (PLT), alanine aminotransferase (ALT), aspartate aminotransferase (AST), albumin (ALB), glucose (Glu), urea, uric acid (UA), creatinine (Cr), triglyceride (TG), total cholesterol (TC), high-density lipoprotein cholesterol (HDL-C), low-density lipoprotein cholesterol (LDL-C), apolipoprotein A (APOA), apolipoprotein B (APOB) and homocysteine (Hcy).

In addition, we evaluated the predictive performance of the model by constructing receptor operating characteristic (ROC) curves and the area under the curve (AUC). At the same time, the net reclassification index (NRI) and integrated discrimination improvement (IDI) were introduced to assess the improvement in model performance. One traditional model (including only the traditional risk factors in model 2) and two new models (including the risk factors in model 2 and either the Phe/Tyr continuous variable or the Phe/Tyr quartile variable) were created by using the logistic regression. SPSS software (version 26.0) and Project R (version 3.6.3) were used for all statistical analyses. *p* < 0.05 was considered statistically significant.

## 3 Results

### 3.1 Study participants and baseline characteristics

**Figure 2:**
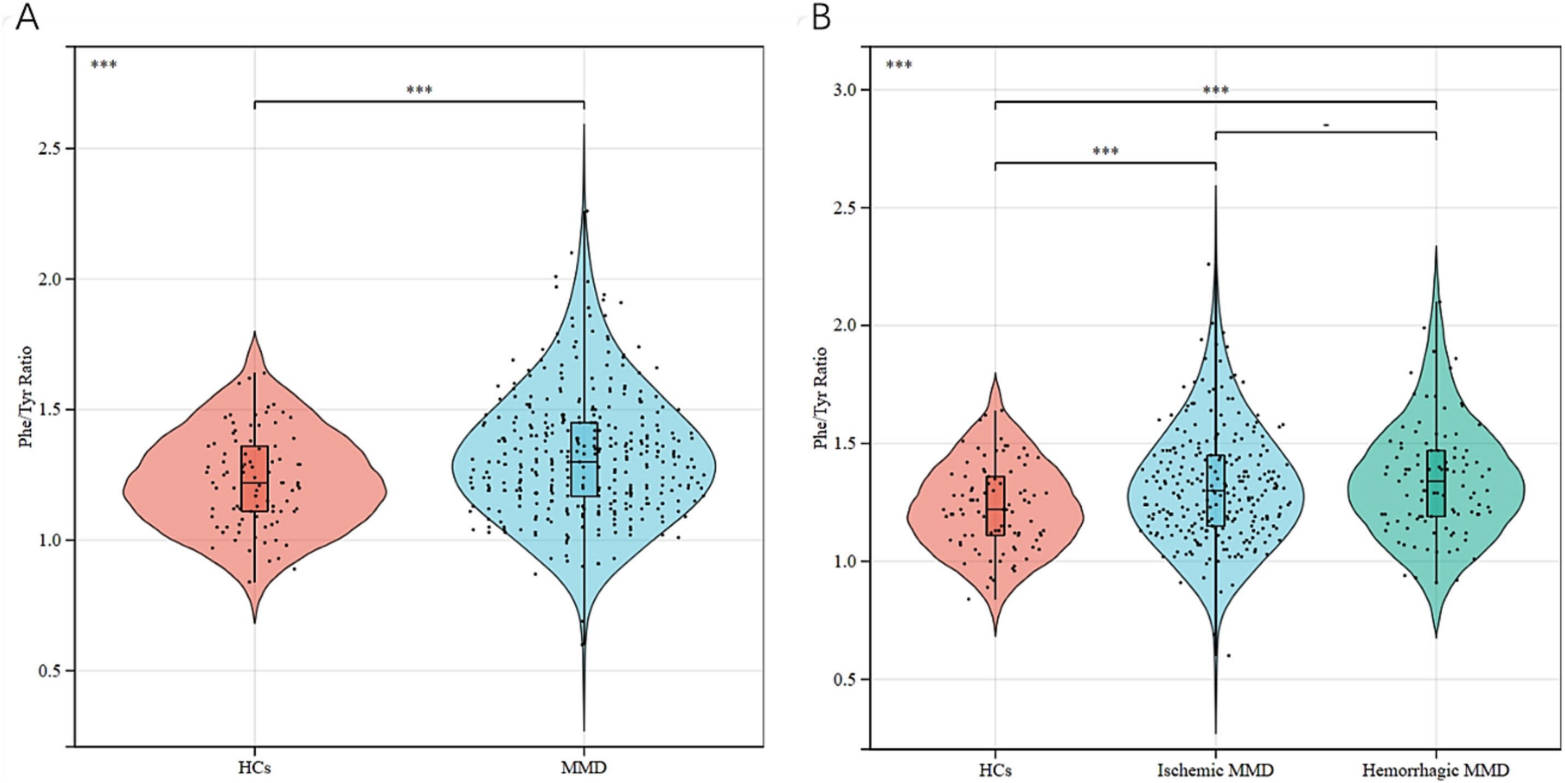
Phe/Tyr ratio between MMD patients and HCs: **(A)** comparison of Phe/Tyr ratio between MMD patients and HCs **(B)** comparison of Phe/Tyr ratio between MMD subtypes and HCs. ^−^*p* > 0.05, ***p ≤ 0.001.

This study included a total of 360 cases of patients with MMD (259 ischemic MMD and 101 hemorrhagic MMD) and 89 cases of HCs. Table 1 shows the baseline comparisons of MMD patients and their subtypes with HCs. Patients with MMD and HCs have no statistical differences in age and sex (*p* > 0.05 for all). MMD patients have higher SBP, DBP, BMI, WBC, NEUT, Glu, ALT, TG and Hcy (*p* < 0.05 for all), while the levels of urea, TC, HDL-C, LDL-C and APOA were lower (*p* < 0.05 for all). MMD patients appear to have more risk factors as they have a higher incidence of hypertension, diabetes, hyperlipidemia, smoking and alcohol consumptions than HCs (*p* < 0.05 for all). The ischemic MMD also showed higher LY and MONO levels than HCs, in addition to the overall trend of differences between MMD and HC. The hemorrhagic MMD maintained the same trend as total MMD only for SBP, WBC, NEUT, Urea, HDL-C and apoA, while apoB was significantly higher than HCs. The serum Phe/Tyr ratio in MMD patients and its subtypes was significantly higher than in HCs (*p* < 0.05 for all) (Figure 2). Phe levels maintained the same trend (*p* < 0.05 for all), while Tyr levels were not different (*p* > 0.05 for all). At the same time, there was no difference in the sum of Phe and Tyr levels in MMD and its subtypes and HCs (*p* > 0.05 for all).

The Phe/Tyr ratio was assessed as quartiles, and the baseline characteristics of MMD patients are shown in Table 2. We found that the serum Phe level increased significantly with higher of Phe/Tyr quartile (the lowest quartile, Q1:86.58 ± 14.18 μmol/L; the highest quartile, Q4:103.97 ± 18.48 μmol/L) (*p* < 0.001). The serum Tyr level was significantly decreased (the lowest quartile, Q1:82.77 ± 14.14 μmol/L; the highest quartile, Q4:65.37 ± 12.90 μmol/L) (*p* < 0.001). Interestingly, the sum of serum Phe and Tyr levels did not differ between quartiles (*p* = 0.763).

**Table 2:** Baseline characteristics of MMD patients according to quartiles of serum Phe/Tyr ratio.

Characteristics	Q1(<1.17, *n* = 90)	Q2 (1.17-<1.31, *n* = 90)	Q3 (1.31-<1.46, *n* = 90)	Q (≥1.46, *n* = 90)	*p*-value
Age, years, mean ± SD	41.79 ± 10.54	43.00 ± 9.48	40.66 ± 10.50	40.96 ± 10.45	0.420
Gender, male, *n* (%)	30 (37.5)	38 (42.7)	45 (48.4)	37 (37.8)	0.399
Medical history					
Hypertension, *n* (%)	28 (31.1)	32 (35.6)	37 (41.1)	34 (37.8)	0.562
Diabetes, *n* (%)	13 (14.4)	11 (14.4)	14 (15.6)	19 (21.1)	0.571
Hyperlipidemia, *n* (%)	11 (12.2)	14 (15.6)	17 (18.9)	12 (13.3)	0.608
Smoking, *n* (%)	19 (21.1)	14 (15.6)	22 (24.4)	16 (17.8)	0.461
Drinking, *n* (%)	7 (7.8)	12 (13.3)	11 (12.2)	12 (13.3)	0.608
Clinical features, mean ± SD					
Heart rate, bpm	78.50 ± 5.70	78.62 ± 6.22	77.76 ± 7.15	79.29 ± 6.52	0.461
SBP, mmHg	132.94 ± 13.87	131.34 ± 10.40	132.90 ± 13.33	132.12 ± 13.48	0.817
DBP, mmHg	81.48 ± 9.68	80.94 ± 8.76	82.96 ± 10.02	82.03 ± 8.83	0.516
BMI, kg/m^2^	25.73 ± 5.43	25.01 ± 4.24	25.78 ± 3.89	25.18 ± 4.34	0.574
RNF213 p.R4810K, *n* (%)					0.170
Wild-type	65 (74.7)	74 (83.1)	74 (87.1)	73 (82.0)	
Mutant	22 (25.3)	15 (16.9)	11 (12.9)	16 (18.0)	
Laboratory examinations, mean ± SD					
WBC count, 10^9^/L	6.90 ± 1.80	7.13 ± 2.27	7.45 ± 2.18	7.20 ± 2.03	0.358
LY count, 10^9^/L	2.04 ± 0.62	1.85 ± 0.56	2.11 ± 0.79	2.02 ± 0.65	0.060
NEUT count, 10^9^/L	4.35 ± 1.55	4.74 ± 2.22	4.77 ± 1.95	4.63 ± 1.97	0.462
MONO count, 10^9^/L	0.37 ± 0.12	0.40 ± 0.18	0.40 ± 0.14	0.36 ± 0.11	0.171
PLT count, 10^9^/L	253.08 ± 84.71	258.63 ± 68.91	249.23 ± 58.28	261.96 ± 69.08	0.631
GLU, mmol/L	5.60 ± 1.60	5.44 ± 1.19	5.44 ± 1.73	5.63 ± 1.65	0.774
ALT, g/L	30.04 ± 32.02	26.35 ± 21.33	27.45 ± 18.23	26.57 ± 23.75	0.728
AST, g/L	20.63 ± 11.04	19.52 ± 9.30	19.37 ± 6.90	18.92 ± 9.39	0.647
ALB, g/L	45.04 ± 2.83	45.08 ± 2.78	45.37 ± 3.13	45.50 ± 3.11	0.682
Urea, mmol/L	4.77 ± 1.19	4.70 ± 1.38	4.60 ± 1.45	4.74 ± 1.47	0.860
Cr, μmol/L	54.02 ± 13.89	57.57 ± 12.58	59.99 ± 15.58	56.61 ± 13.56	0.039
UA, μmol/L	312.39 ± 86.91	310.65 ± 80.47	326.88 ± 98.76	312.57 ± 94.25	0.597
TG, mmol/L	1.41 ± 1.13	1.34 ± 0.84	1.60 ± 1.39	1.36 ± 0.76	0.334
TC, mmol/L	4.20 ± 1.12	4.19 ± 1.12	4.31 ± 1.00	4.21 ± 0.87	0.813
HDL-C, mmol/L	1.31 ± 0.30	1.34 ± 0.27	1.29 ± 0.28	1.31 ± 0.28	0.679
LDL-C, mmol/L	2.39 ± 0.96	2.40 ± 0.76	2.51 ± 0.86	2.45 ± 0.79	0.784
ApoA, g/L	1.32 ± 0.27	1.29 ± 0.25	1.28 ± 0.23	1.30 ± 0.25	0.741
ApoB, g/L	0.82 ± 0.24	0.82 ± 0.21	0.86 ± 0.23	0.86 ± 0.21	0.343
Homocysteine, μmol/L	12.72 ± 6.22	13.90 ± 7.58	13.72 ± 8.56	13.24 ± 4.79	0.668
Phenylalanine, μmol/L	88.00 ± 14.11	93.20 ± 16.38	97.44 ± 18.03	103.80 ± 19.02	<0.001
Tyrosine, μmol/L	83.16 ± 14.48	75.96 ± 13.59	70.89 ± 12.84	64.08 ± 12.86	<0.001
Phe/Tyr ratio	1.06 ± 0.09	1.23 ± 0.04	1.37 ± 0.04	1.63 ± 0.17	<0.001
Phe + Tyr, mmol/L	171.16 ± 27.04	169.16 ± 29.83	168.34 ± 30.77	167.88 ± 30.95	0.887
Clinical type					0.591
Ischemic type	69 (76.7)	62 (68.9)	66 (73.3)	62 (68.9)	
Hemorrhagic type	21 (23.3)	28 (31.1)	24 (26.7)	28 (31.1)	
Admission mRS, *n* (%)					0.499
0–2	82 (91.1)	81 (90.0)	84 (93.3)	78 (86.7)	
3–5	8 (8.9)	9 (10.0)	6 (6.7)	12 (13.3)	
Suzuki stage, *n* (%)					0.311
1–2	25 (27.8)	26 (28.9)	20 (22.2)	30 (33.3)	
3–4	42 (46.7)	46 (51.1)	55 (61.1)	39 (43.3)	
5–6	23 (25.6)	18 (20.0)	15 (16.7)	21 (23.3)	

### 3.2 Clinical features of the RNF213 p.R4810K variant

The enrolled patients were stratified into mutation and no mutation groups based on the presence of the RNF213 p.R4810K variant. Analysis of the Phe/Tyr ratio revealed no significant differences between the mutation (1.30 ± 0.24) and no mutation (1.33 ± 0.23) groups when treated as a continuous variable (*p* = 0.379). Similarly, categorization into quartiles (Q1–Q4) showed no association with mutation status (*p* = 0.17). Functional status at admission, assessed using the modified Rankin Scale (mRS), also demonstrated no significant disparity between groups when dichotomized as high (mRS 0–2) versus low (mRS 3–5) (*p* = 0.133). In contrast, a significant association was observed between the p.R4810K variant and advanced Suzuki stages (*p* = 0.023). Mutation patients exhibited a higher proportion of advanced-stage disease (Suzuki 3–4: 11.1%; Suzuki 5–6: 4.6%) compared to the no mutation group (Suzuki 3–4: 39.7%; Suzuki 5–6: 16.6%) (Supplementary Table S2).

### 3.3 Association of Phe/Tyr with the risk of MMD

**Figure 3:**
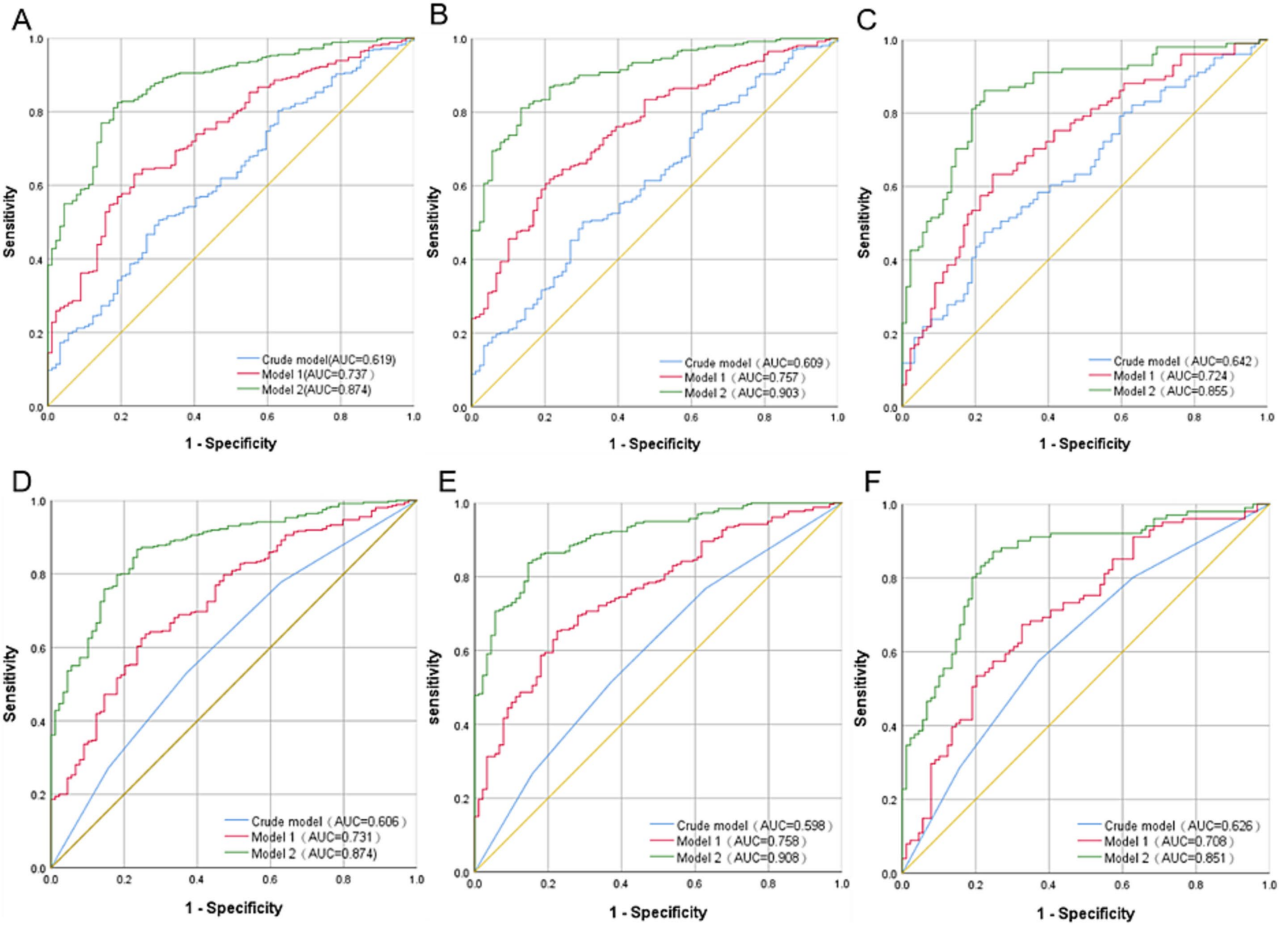
Comparison of ROC curves of Phe/Tyr ratio for the risk of MMD and its subtypes in different models: **(A)** MMD overall; **(B)** ischemic MMD; and **(C)** hemorrhagic MMD; Comparison of ROC curves of Phe/Tyr ratio quartiles for the risk of MMD and its subtypes in different models. **(D)** MMD overall; **(E)** Ischemic MMD; **(F)** Hemorrhagic MMD.

Table 3 presents the association between the serum Phe/Tyr ratio and the risk of MMD. We found that Phe/Tyr was positively correlated with the risk of MMD in the crude model (OR: 8.938, 95%CI:2.715–29.421, *p* < 0.001). After model adjustment, the risk of MMD also increased with Phe/Tyr in model 1(OR: 10.698, 95%CI: 2.947–38.839, p < 0.001) and model 2 (OR: 14.035, 95%CI: 2.784–70.748, *p* = 0.001). In addition, The ROC curves showed that model 2 (AUC = 0.874) had significantly better predictive accuracy than the crude model (AUC = 0.619) and model 1 (AUC = 0.737) (Figure 3A).

**Table 3:** Association of Phe/Tyr with the risk of MMD and its subtypes.

Phe/Tyr	No. of	Crude Model		Model 1		Model 2	
	Events (%)	OR (95% CI)	*p*-value	OR (95% CI)	*p*-value	OR (95% CI)	*p*-value
Moyamoya overall continuous	360 (80.2)	8.938 (2.715,29.421)	<0.001	10.698 (2.947,38.839)	<0.001	14.035 (2.784,70.748)	0.001
Quartiles							
Q1 (<1.15)	80 (70.8)	1.0 (Ref.)		1.0 (Ref.)		1.0 (Ref.)	
Q2 (1.15 to <1.30)	89 (79.5)	1.596 (0.866,2.944)	0.134	1.932 (1.008,3.703)	0.047	2.181 (1.008,4.717)	0.048
Q3 (1.30 to <1.45)	93 (83.0)	2.019 (1.066,3.824)	0.031	2.370 (1.197,4.692)	0.013	2.789 (1.208,6.436)	0.016
Q4 (> = 1.45)	98 (87.5)	2.887 (1.446,5.765)	0.003	3.214 (1.539,6.714)	0.002	3.531 (1.468,8.494)	0.005
Ischemic MMD continuous	259 (74.4)	7.735 (2.248,26.616)	0.001	7.194 (1.801,28.726)	0.005	18.658 (2.502,139.108)	0.004
Quartiles							
Q1 (<1.15)	60 (64.5)	1.0 (Ref.)		1.0 (Ref.)		1.0 (Ref.)	
Q2 (1.15 to <1.30)	66 (74.2)	1.578 (0.835,2.984)	0.160	2.36 (1.167,4.771)	0.017	5.419 (2.074,14.16)	0.001
Q3 (1.30 to <1.45)	64 (77.1)	1.853 (0.952,3.604)	0.069	2.154 (1.025,4.523)	0.043	3.367 (1.254,9.039)	0.016
Q4 (> = 1.45)	69 (83.1)	2.711 (1.327,5.538)	0.006	2.577 (1.186,5.600)	0.017	4.635 (1.602,13.408)	0.005
Hemorrhagic MMD continuous	101 (53.2)	15.870 (3.474,72.493)	<0.001	27.418 (5.069,148.3)	<0.001	23.028 (2.859,185.479)	0.003
Quartiles							
Q1 (<1.15)	20 (37.7)	1.0 (Ref.)		1.0 (Ref.)		1.0 (Ref.)	
Q2 (1.15 to <1.30)	23 (50.0)	1.625 (0.740,3.678)	0.221	1.800 (0.773,4.193)	0.173	1.598 (0.539,4.731)	0.398
Q3 (1.30 to <1.45)	29 (60.4)	2.518 (1.129,5.616)	0.024	3.244 (1.371,7.675)	0.007	2.597 (0.875,7.704)	0.086
Q4 (> = 1.45)	29 (67.4)	3.418 (1.467,7.963)	0.004	4.309 (1.726,10.758)	0.002	3.834 (1.261,11.659)	0.018

**Figure 4:**
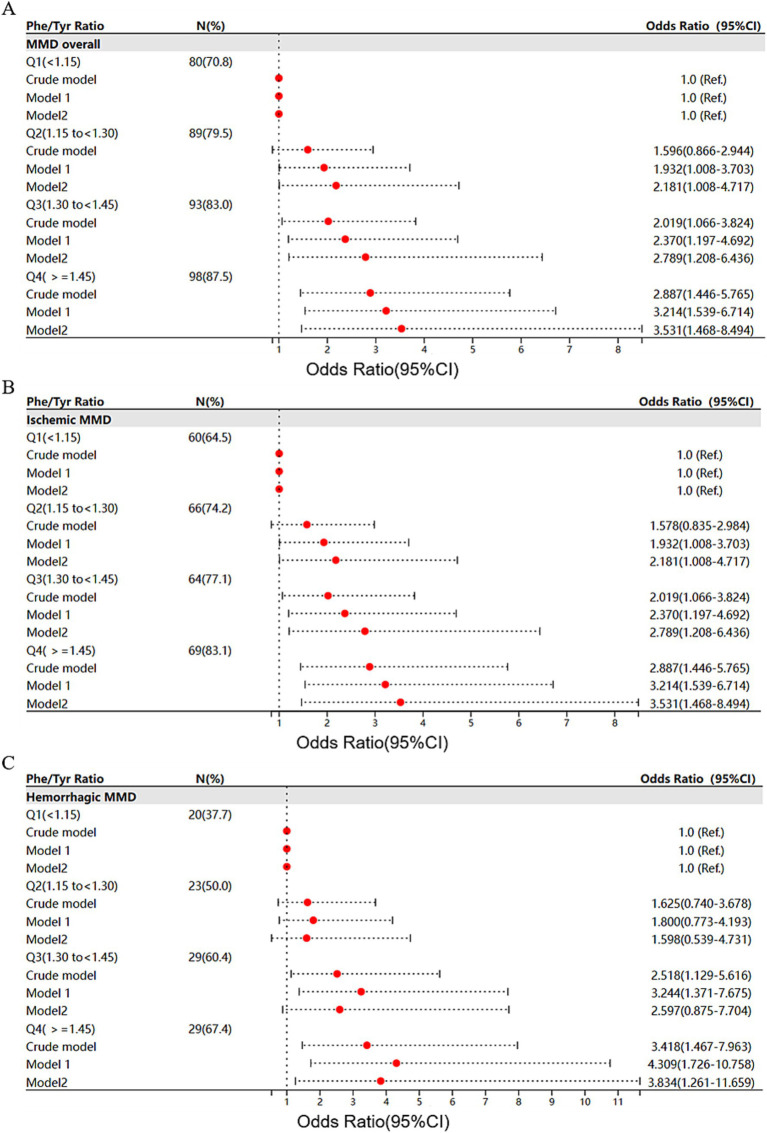
Forest plots of Phe/Tyr association with risk of MMD and its subtypes: **(A)** MMD overall; **(B)** ischemic MMD; and **(C)** hemorrhagic MMD.

When Phe/Tyr ratio was assessed as quartiles, we found that the proportion of total MMD events increased with increasing quartile of Phe/Tyr ratio. Phe/Tyr had higher risk of MMD in third (Q3) and fourth quartile (Q4) in the crude model compared to first quartile (Q1) (Q3, OR: 2.019, 95%CI:1.066–3.824, *p* = 0.031; Q4, OR: 2.887, 95%CI:1.446–5.765, *p* = 0.003). The risk of MMD increased with increasing Phe/Tyr quartile in model 1 (Q3, OR: 2.37, 95%CI:1.197–4.692, *p* = 0.013; Q4, OR: 3.214, 95%CI:1.539–6.714, *p* = 0.002). Model 2 also follows the same trend (Q3, OR: 2.789, 95%CI:1.208–6.436, *p* = 0.016; Q4, OR: 3.531, 95%CI:1.468–8.494, *p* = 0.005) (Figure 4A). Similarly, the ROC curve shows that Model 2 (AUC = 0.872) has significantly higher predictive power than the crude model (AUC = 0.606) and model 1(AUC = 0.731) (Figure 3D).

### 3.4 Association of Phe/Tyr with the risk of MMD subtypes

The MMD subtypes also followed the same trend as the overall. The risk of ischemic MMD increased as the ratio of Phe/Tyr increased in the crude model (OR: 7.735, 95%CI: 2.248–26.616, *p* = 0.001), model 1 (OR: 7.194, 95%CI: 1.801–28.726, *p* = 0.005) and model 2 (OR: 18.658, 95%CI: 2.502–139.108, *p* = 0.004). The AUC of the ROC curve increased with the adjustment of the model (crude model, AUC = 0.609; model 1, AUC = 0.757; model 2, AUC = 0.903, Figure 3B). When Phe/Tyr was evaluated as quartiles, the risk of ischemic MMD was significantly higher in the higher quartile in the crude model (Q4, OR:2.711, 95%CI:1.327–5.538, *p* = 0.006), model 1 (Q4, OR:2.577, 95%CI:1.186–5.600, *p* = 0.017) and model 2 (Q4, OR: 4.635, 95%CI,1.602–13.408, *p* = 0.005) (Figure 4B). The ROC curve shows that model 2 (AUC = 0.908) has better predictive power than the crude model (AUC = 0.598) and model 1 (AUC = 0.758) (Figure 3E).

The risk of hemorrhagic MMD increased as the ratio of Phe/Tyr increased in the crude model (OR: 15.87, 95%CI: 3.474–72.493, *p* < 0.001), model 1 (OR: 27.418, 95%CI: 5.099–148.3, *p* < 0.001) and model 2 (OR: 23.028, 95%CI: 2.859–185.479, *p* = 0.003). The AUC value of the ROC curve increased with model development (crude model, AUC = 0.642; for model 1, AUC = 0.724; model 2, AUC = 0.855) (Figure 3C). When Phe/Tyr was evaluated as quartiles, the risk of hemorrhagic MMD was significantly higher in the higher quartile in the crude model (Q4, OR:3.418, 95%CI: 1.467–7.963, *p* = 0.004), model 1 (Q4, OR = 4.309, 95%CI: 1.726,10.758, *p* = 0.002) and model 2 [3.834 (1.261,11.659)] (Figure 4C). The AUC curve shows that model 2 (AUC = 0.851) has a better predictive power than the crude model (AUC = 0.626) and model 1 (AUC = 0.708) (Figure 3F).

### 3.5 Clinical correlations between Phe/Tyr and MMD

We investigated the association between imaging findings and the Phe/Tyr ratio in all patients. Suzuki staging was categorized based on the higher grade observed bilaterally: grade 1 (Suzuki stage 1–2), grade 2 (Suzuki stage 3–4), and grade 3 (Suzuki stage 5–6). No significant differences in Phe/Tyr levels were observed across Suzuki stages (*p* = 0.686). Additionally, preoperative mRS scores were used to stratify patients into two groups: a high-functioning group (mRS ≤ 2) and a low-functioning group (3 ≤ mRS ≤ 5). The Phe/Tyr ratio did not differ significantly between these groups (1.32 ± 0.23 vs. 1.35 ± 0.27, *p* = 0.552) (Supplementary Table S3).

### 3.6 Incremental predictive value of Phe/Tyr for the risk of MMD

We investigated whether the ability to predict the risk of overall MMD and its subtypes could be improved by adding Phe/Tyr to the base model (including all risk factors in model 2). The risk reclassification was significantly improved with the addition of Phe/Tyr to the conventional risk factors for overall MMD (NRI: 39.3%, *p* < 0.001; IDI: 2.5%, *p* = 0.012), ischemic MMD (NRI: 39.6%, *p* = 0.001; IDI: 2.0%, *p* = 0.039) and hemorrhagic MMD (NRI: 50.0%, p < 0.001: IDI: 4.3%, *p* = 0.003). The risk reclassification was also significantly improved with the addition of Phe/Tyr quartiles to the conventional risk factors overall MMD (NRI: 38.6%, *p* < 0.001; IDI: 0.2%, *p* = 0.025), ischemic MMD (NRI: 36.4%, *p* = 0.003) and hemorrhagic MMD (NRI: 48.9%, *p* < 0.001: IDI: 3.0%, *p* = 0.013) (Table 4).

**Table 4:** Improvement of model predictivity by addition of Phe/Tyr.

		NRI		IDI	
Variables	AUC	Estimate(95%CI)	*p*-value	Estimate(95%CI)	*p*-value
MMD overall					
Basic model	0.863	Ref.		Ref.	
Basic model + Phe/Tyr continuous	0.874	0.393 (0.167–0.617)	<0.001	0.025 (0.005–0.044)	0.012
Basic model + Phe/Tyr quartiles	0.874	0.386 (0.161–0.612)	<0.001	0.002(0.003–0.039)	0.025
Ischemic MMD					
Basic model	0.895	Ref.		Ref.	
Basic model + Phe/Tyr continuous	0.903	0.396 (0.159–0.632)	0.001	0.020 (0.001–0.039)	0.039
Basic model + Phe/Tyr quartiles	0.908	0.364 (0.128–0.600)	0.003	0.018 (0.000–0.036)	0.055
Hemorrhagic MMD					
Basic model	0.835	Ref.		Ref.	
Basic model + Phe/Tyr continuous	0.855	0.500 (0.226–0.773)	<0.001	0.043 (0.015–0.071)	0.003
Basic model + Phe/Tyr quartiles	0.851	0.489 (0.213–0.765)	<0.001	0.030 (0.006–0.053)	0.013

## 4 Discussion

In this study, we found that Phe/Tyr levels were significantly higher in MMD patients than HCs. Meanwhile, we further studied the relationship between Phe/Tyr and the risk of MMD by using regression models, and found that high Phe/Tyr could increase the risk of MMD. Both ischemic MMD and hemorrhagic MMD showed good fit to the model. After adjusting the baseline characteristics and laboratory detection indicators, the clinical regression model further confirmed that Phe/Tyr was an independent risk factor for MMD, and the ROC curve was used to verify the accuracy of the model prediction. The inclusion of Phe/Tyr in the model provided significant improvements in risk reclassification and identification of MMD and its subtypes. These findings validate the value of Phe/Tyr as a novel biomarker for MMD, and to our knowledge, this is the largest and first study to assess the association of Phe/Tyr with the risk of MMD.

Phe is an essential amino acid that plays a crucial role in the biosynthesis of a wide range of cells and tissues. Its main metabolic enzymes are phenylalanine hydroxylase (PAH), converting it into Tyr, and glutamate oxaloacetate transaminase 1(GOT 1), converting it into phenylpyruvic acid (14, 15). Tyr is an important precursor substance for the synthesis of a number of neurotransmitters and hormones, including dopamine, epinephrine and norepinephrine (catecholamine), and also plays an important role in regulating mood and protecting the nervous system (16). In recent years, many studies have demonstrated that the overexpression of Phe was present in several central nervous system disorders, such as Alzheimer’s disease (10) and stroke (13). Serum phenylpyruvic acid levels have been shown to be reduced in patients with MMD. However, it is worth noting that N-acetyl-l-tyrosine, a key intermediate in catecholamine synthesis, showed a significant increase in MMD (17). Our study showed that Phe/Tyr levels were significantly higher in MMD patients than in HCs. Additionally, we observed a positive correlation between Phe/Tyr levels and the risk of MMD.

Phe/Tyr was considered a biomarker for some inflammatory responses, and Phe/Tyr showed numerical differences in different disease conditions, such as 1.24 in the study of patients with trauma and sepsis (18), 1.47 in patients with ovarian cancer (19), 1.75 in acute ischemic stroke (13), and 1.33 in this study. While the elevation of the Phe/Tyr ratio may not be specific to MMD, the significantly higher levels observed in MMD patients compared to HCs likely reflect unique metabolic dysregulation mechanisms. Chronic cerebral ischemia in MMD could result in diminished phenylalanine hydroxylase activity, thereby disrupting phenylalanine metabolism and contributing to the observed biomarker profile. In ovarian carcinoma, tumor inflammation and immune activation can interfere with the conversion of phenylalanine by attenuating PAH activity, leading to an increase in phenylalanine concentration (19). Furthermore, higher Phe/Tyr ratio associated with immune activation and inflammation in cardiovascular disease patients (20). Although he reasons for the elevated Phe/Tyr in patients with MMD are speculative, we have reason to suspect that this phenomenon is also present in MMD. Multiple studies have suggested that the progression of MMD is associated with inflammation (21). Tetrahydrobiopterin (BH4), a cofactor for PAH, was susceptible to significant depletion in the inflammatory response, leading to inhibition of Phe metabolism (22). Therefore, higher Phe/Tyr levels indicate that the pathway by which PAH converts Phe to Tyr may be impaired. Our study also confirmed this: with increasing Phe/Tyr quartile, serum Phe concentration gradually increased and Tyr gradually decreased. As a precursor of catecholamines, Tyr is mainly converted in the adrenal medulla. The reason for the decrease in serum Tyr concentration may be that Tyr is actively converted to catecholamine. In addition, inflammatory response in acute ischemic stroke could lead to increased oxidation of Tyr (23). Cerebrovascular diseases were thought to trigger pronounced catecholamine surges through diverse ways (24) It has been reported that increased catecholamine secretion may play a key role in the onset and progression of MMD (17, 25).

Elevated Phe/Tyr levels increase the risk of MMD. Although no significant association was observed between Phe/Tyr levels and Suzuki staging, this does not preclude its potential involvement in pathological vascular proliferation or disease progression in MMD. Phe/Tyr elevation may still play a role in endothelial dysfunction or collateral vessel formation, independent of the angiographic stage. Simultaneously, accumulating and metabolizing phenylalanine in the brain following a brain injury can lead to more severe brain damage and accelerate disease progression (16). Therefore, the metabolism of Phe after MMD is being analyzed in order to find reasonable methods improve the accumulation of Phe. In the current background of revascularization as the main basic treatment, it provides a new treatment plan. A study revealed a significant increase in Phe levels in the para-infarct cortex of experimental ischemic stroke mice, and hydroxysafflor yellow A could show a strong brain protective effect on ischemic stroke by reducing the change of Phe level induced by ischemic stress (26). MMD is a chronic hypoxic–ischemic cerebrovascular disease. Our study has confirmed that there is a significant increase in Phe levels in MMD. Another study found that the accumulation of Phe caused by exogenous addition could promote endothelial cell apoptosis. Meanwhile, Medioresinol (MDN) promoted Phe metabolism by increasing the expression of GOT1 and PAH after cerebral ischemia, and inhibited the apoptosis of cerebral microvascular endothelial cells (16). Abnormalities in apoptosis are widespread in vascular endothelial cells in MMD and apoptosis may play an important role in the development of MMD (27). It is reasonable to assume that the progression of inflammation and cerebral ischemia during MMD episodes affects PAH activity thus leading to inhibition of Phe metabolism. BH4 has been approved for the treatment of phenylketonuria due to congenital PAH deficiency (28). Although BH4 was not measured in this study, previous studies have demonstrated the plausibility of promoting the conversion of Phe through exogenous supplementation of BH4 after MMD to attenuate brain damage. Meanwhile, cerebral ischemic state promotes the synthesis and secretion of catecholamines *in vivo*, depleting tyrosine in the process and contributing to the elevated Phe/Tyr ratio in MMD patients. However, amino acid metabolism is systemic and multifactorial. Although our study confirmed the plausibility of Phe/Tyr as an independent risk factor for MMD. The lack of association with Suzuki staging or mRS scores may reflect the complexity of MMD progression, where metabolic disturbances and vascular remodeling operate through divergent pathways. Additionally, combining Phe/Tyr with other inflammatory or hemodynamic markers may enhance its predictive value for identifying high-risk subgroups or monitoring therapeutic responses.

This study still has some limitations. Firstly, the patients included in this study were all from one neurosurgery center, which may require a large multicenter prospective study to reduce bias; secondly, the study subjects were all Chinese adults, and it is uncertain whether children or other ethnic groups would have the same result; thirdly, the patients’ dietary intake could not be followed up. Different dietary habits may also cause differences in the distribution of amino acids. Fourthly, this is a cross-sectional study and it is not possible to analyze behavior over time or to establish long-term trends, making it difficult to establish a causal relationship between Phe/Tyr and MMD. Fifthly, the current investigation was not designed to evaluate potential associations between the Phe/Tyr and postoperative disease progression or longitudinal clinical outcomes. Prospective, longitudinal studies incorporating comprehensive clinical follow-up data are warranted to rigorously assess the prognostic significance of this biochemical parameter in postoperative monitoring paradigms.

## 5 Conclusion

Serum Phe/Tyr ratio is associated with the risk of MMD and its subtypes, showing its potential as a potential biomarker for MMD.

## Data Availability

The datasets presented in this study can be found in online repositories. The names of the repository/repositories and accession number(s) can be found in the article/Supplementary material.

## References

[1] Kuroda S Houkin K. Moyamoya disease: current concepts and future perspectives. Lancet Neurol. (2008) 7:1056–66. doi: 10.1016/S1474-4422(08)70240-0, PMID: 18940695

[2] Huang S Guo ZN Shi M Yang Y Rao M. Etiology and pathogenesis of Moyamoya disease: an update on disease prevalence. Int J Stroke. (2017) 12:246–53. doi: 10.1177/1747493017694393, PMID: 28381201

[3] Ahn HS Kazmi SZ Kang T Kim DS Kim HJ. Familial risk for Moyamoya disease among first-degree relatives, based on a population-based aggregation study in Korea. Stroke. (2020) 51:2752–60. doi: 10.1161/STROKEAHA.120.029251, PMID: 32811391

[4] Ihara M Yamamoto Y Hattori Y Liu W Kobayashi H Ishiyama H. Moyamoya disease: diagnosis and interventions. Lancet Neurol. (2022) 21:747–58. doi: 10.1016/s1474-4422(22)00165-X, PMID: 35605621

[5] Kamada F Aoki Y Narisawa A Abe Y Komatsuzaki S Kikuchi A. A genome-wide association study identifies RNF213 as the first Moyamoya disease gene. J Hum Genet. (2011) 56:34–40. doi: 10.1038/jhg.2010.132, PMID: 21048783

[6] Liu W Morito D Takashima S Mineharu Y Kobayashi H Hitomi T. Identification of RNF213 as a susceptibility gene for moyamoya disease and its possible role in vascular development. PLoS One. (2011) 6:e22542. doi: 10.1371/journal.pone.0022542, PMID: 21799892 PMC3140517

[7] Ge P Zhang Q Ye X Liu X Deng X Wang J. Modifiable risk factors associated with Moyamoya disease: a case-control study. Stroke. (2020) 51:2472–9. doi: 10.1161/STROKEAHA.120.030027, PMID: 32640948

[8] Mertens R Graupera G-MM i Gerhardt H Bersano A Tournier-Lasserve E Mensah M. The genetic basis of Moyamoya disease. Transl Stroke Res. (2022) 13:25–45. doi: 10.1007/s12975-021-00940-2, PMID: 34529262 PMC8766392

[9] Chen W-S Wang C-H Cheng C-W Liu M-H Chu C-M Wu H-P. Elevated plasma phenylalanine predicts mortality in critical patients with heart failure. Esc Heart Fail. (2020) 7:2884–93. doi: 10.1002/ehf2.12896, PMID: 32618142 PMC7524095

[10] Wang X Sun G Feng T Zhang J Huang X Wang T. Sodium oligomannate therapeutically remodels gut microbiota and suppresses gut bacterial amino acids-shaped neuroinflammation to inhibit Alzheimer's disease progression. Cell Res. (2019) 29:787–803. doi: 10.1038/s41422-019-0216-x, PMID: 31488882 PMC6796854

[11] Kopple J. Phenylalanine and tyrosine metabolism in chronic kidney failure. J Nutr. (2007) 137:1586S–90S. doi: 10.1093/jn/137.6.1586S, PMID: 17513431

[12] Hirayama M Tsunoda M Yamamoto M Tsuda T Ohno K. Serum tyrosine-to-phenylalanine ratio is low in Parkinson's disease. J Parkinson Dis. (2016) 6:423–31. doi: 10.3233/JPD-150736, PMID: 27061063

[13] Ormstad H Verkerk R Sandvik L. Serum phenylalanine, tyrosine, and their ratio in acute ischemic stroke: on the trail of a biomarker? J Mol Neurosci. (2016) 58:102–8. doi: 10.1007/s12031-015-0659-6, PMID: 26423306

[14] Flydal MI Aurora M. Phenylalanine hydroxylase: function, structure, and regulation. IUBMB Life. (2013) 65:341–9. doi: 10.1002/iub.1150, PMID: 23457044

[15] van-Spronsen F Smit P Koch R. Phenylketonuria: tyrosine beyond the phenylalanine-restricted diet. J Inherit Metab Dis. (2001) 24:1–4. doi: 10.1023/A:1005689232358, PMID: 11286377

[16] Wang Y Guan X Gao CL Ruan W Pang T. Medioresinol as a novel PGC-1α activator prevents pyroptosis of endothelial cells in ischemic stroke through PPARα-GOT1 axis. Pharmacol Res. (2021) 169:105640. doi: 10.1016/j.phrs.2021.105640, PMID: 33915296

[17] Guo Q Wang QN Li J Liu S Wang X Yu D. Proteomic and metabolomic characterizations of moyamoya disease patient sera. Brain Behav. (2023) 13:e3328. doi: 10.1002/brb3.3328, PMID: 37962021 PMC10726768

[18] Ploder M Neurauter G Spittler A Schroecksnadel K Roth E Fuchs D. Serum phenylalanine in patients post trauma and with sepsis correlate to neopterin concentration. Amino Acids. (2008) 35:303–7. doi: 10.1007/s00726-007-0625-x, PMID: 18163176

[19] Neurauter G Grahmann A Klieber M Zeimet A Ledochowski M Sperner-Unterweger B. Serum phenylalanine concentrations in patients with ovarian carcinoma correlate with concentrations of immune activation markers and of isoprostane. Cancer Lett. (2008) 272:141–7. doi: 10.1016/j.canlet.2008.07.002, PMID: 18701209

[20] Murr C Grammer TB Meinitzer A Kleber ME März W. Immune activation and inflammation in patients with cardiovascular disease are associated with higher phenylalanine to tyrosine ratios: the ludwigshafen risk and cardiovascular health study. J Amino Acids. (2014) 2014:783730. doi: 10.1155/2014/783730, PMID: 24660059 PMC3934657

[21] Mikami T Suzuki H Komatsu K Mikuni N. Influence of inflammatory disease on the pathophysiology of Moyamoya disease and quasi-moyamoya disease. Neurol Med-chir. (2019) 59:361–70. doi: 10.2176/nmc.ra.2019-0059, PMID: 31281171 PMC6796064

[22] Kim HK Han J. Tetrahydrobiopterin in energy metabolism and metabolic diseases. Pharmacol Res. (2020) 157:104827. doi: 10.1016/j.phrs.2020.104827, PMID: 32348841

[23] Ormstad H Verkerk R Aass H Amthor K Sandvik L. Inflammation-induced catabolism of tryptophan and tyrosine in acute ischemic stroke. J Mol Neurosci. (2013) 51:893–902. doi: 10.1007/s12031-013-0097-2, PMID: 23990339

[24] Du Y Demillard LJ Ren J. Catecholamine-induced cardiotoxicity: a critical element in the pathophysiology of stroke-induced heart injury. Life Sci. (2021) 287:120106. doi: 10.1016/j.lfs.2021.120106, PMID: 34756930

[25] Fumihiro M Yasuo M Atsushi W Shirokane K Igarashi T Shimizu K. Case report: a case of Moyamoya syndrome associated with multiple endocrine neoplasia type 2A. Front Endocrinol. (2021) 12:703410. doi: 10.3389/fendo.2021.703410, PMID: 34858321 PMC8632216

[26] Suning C Mao S Xianghui Z Zhifu Y Wenxing L. Neuroprotection of hydroxysafflor yellow a in experimental cerebral ischemia/reperfusion injury via metabolic inhibition of phenylalanine and mitochondrial biogenesis. Mol Med Rep. (2019) 19:3009–20. doi: 10.3892/mmr.2019.9959, PMID: 30816517 PMC6423596

[27] Takagi Y Kikuta KI Sadamasa N Nozaki K Hashimoto N. Caspase-3-dependent apoptosis in middle cerebral arteries in patients with moyamoya disease. Neurosurgery. (2006) 59:894–900. doi: 10.1227/01.NEU.0000232771.80339.15, PMID: 17038954

[28] Wilson SK Thomas J. BH4 as a therapeutic target for ADHD: relevance to neurotransmitters and stress-driven symptoms. J Atten Disord. (2023) 28:161–7. doi: 10.1177/10870547231204012, PMID: 37942650

